# Follicular dendritic cell sarcoma of the neck: Report of a case treated by surgical excision and COP plus (PEG)-liposomal doxorubicin

**DOI:** 10.1186/1756-9966-27-33

**Published:** 2008-09-02

**Authors:** Francesco Pisani, Mirella Marino, Steno Sentinelli, Maria Concetta Petti

**Affiliations:** 1 Department of Hematology, Regina Elena National Cancer Institute, Rome, Italy; 2 Department of Phatology, Regina Elena National Cancer Institute, Rome, Italy

## Abstract

**Background:**

Follicular dendritic cell (FDC) sarcoma is a rare neoplasm arising in lymph nodes but also in extranodal sites from accessory cells of the immune system that are essential for the function of antigen presentation and germinal center reaction regulation. FDC sarcoma has a significant recurrent and metastatic potential and for these reason it should be viewed as an intermediate grade malignancy.

**Methods:**

We report the case of a 49-year old woman patient who showed persistent, enlarged, hard, cervical lymph node. The most common histologic feature was the presence of oval to spindle cells with elongated nuclei, vesicular or stippled chromatin and scant eosinophilic cytoplasm. Immunohistochemically, tumor cells were diffusely positive for follicular dendritic cell markers CD21, CD23 and negative for cytokeratin.

The patient after complete excision of the lymph node underwent five courses of adjuvant chemotherapy with COP plus PEG-liposomal doxorubicin, considering the propensity of the tumor to metastasize.

**Results:**

No hematological or cardiac toxicity were registered and among the other extra hematological effects only transitory palmar erythrodysesthesia is worthy of mention. After a follow up of 5 years the patient is alive and in CR.

**Conclusion:**

These results suggest that this therapeutic modality may be useful in the management of FDC sarcoma.

## Background

Follicular dendritic cell sarcoma is a rare tumor arising from antigen-presenting cells of the B-cell follicles of nodal and extranodal sites; these cells are important for germinal center reaction regulation. It was first described in 1986 by Monda et al [1] and in recent years there has been an increasing interest in this neoplasm due to availability of specific antibodies to confirm FDC lineage and thus wider recognition.

Almost all patients have been adults, with a median age of 40 years and with a slight female predominance [2]. Most patients present with cervical or axillary lymphoadenopathy but extranodal sites, including oral cavity, tonsil, gastrointestinal tract, soft tissue [3] and breast, may occur in almost one third of the patients.

We report a case of nodal FDC sarcoma who was successfully diagnosed and treated with surgery and chemotherapy, comprehending PEG-liposomal doxorubicin.

## Methods

A 49 year old woman presented at our Institute with an enlarged (2 cm), hard, mobile lymph node of the right cervical area that had been present for about 3 months.

An ultrasound examination of the thyroid gland showed two nodules of 12 × 8 mm and 6 × 6 mm and a fine needle aspiration cytology was benign. The patient underwent a right neck dissection with complete excision of lymph node and multiple biopsies of nasopharynx, pyriform sinus and postcricoid area.

The fiberlaryngoscopic and oralpharyngealscopic evaluations were negative for extensive or focal malignancies.

### Histopathologic findings

**Figure 1:**
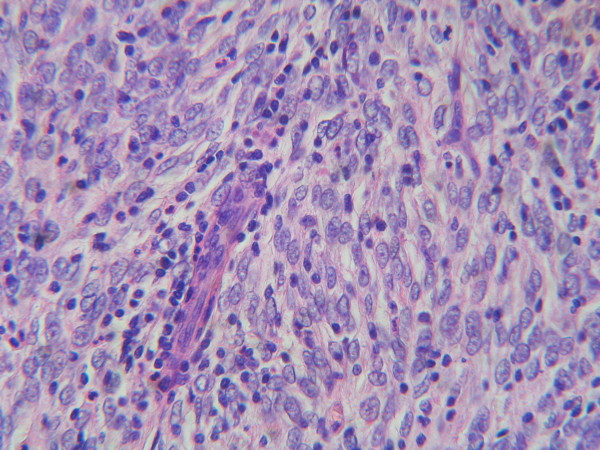
**Partial substitution of lymph node from a proliferation of oval-to-spindle cells with vesicular or stippled chromatin, elongated nuclei, scant eosinophilic cytoplasm and with low mitotic rate.** H&E, × 400.

The 2 cm lymph node was partially substituted from a proliferation of oval-to-spindle cells with vesicular or stippled chromatin and oval to spindle shaped nuclei. The cells had scant eosinophilic cytoplasm with indistinct border and the mitotic rate was low (Figure 1).

**Figure 2:**
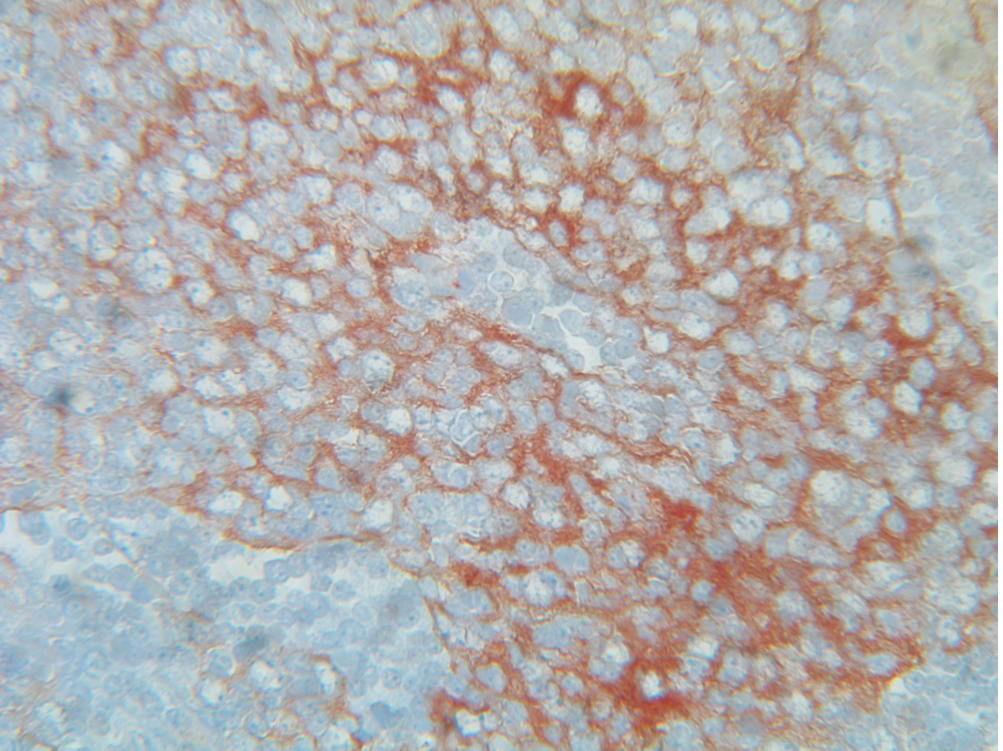
Immunohistochemical staining: Positive staining for CD 21

**Figure 3:**
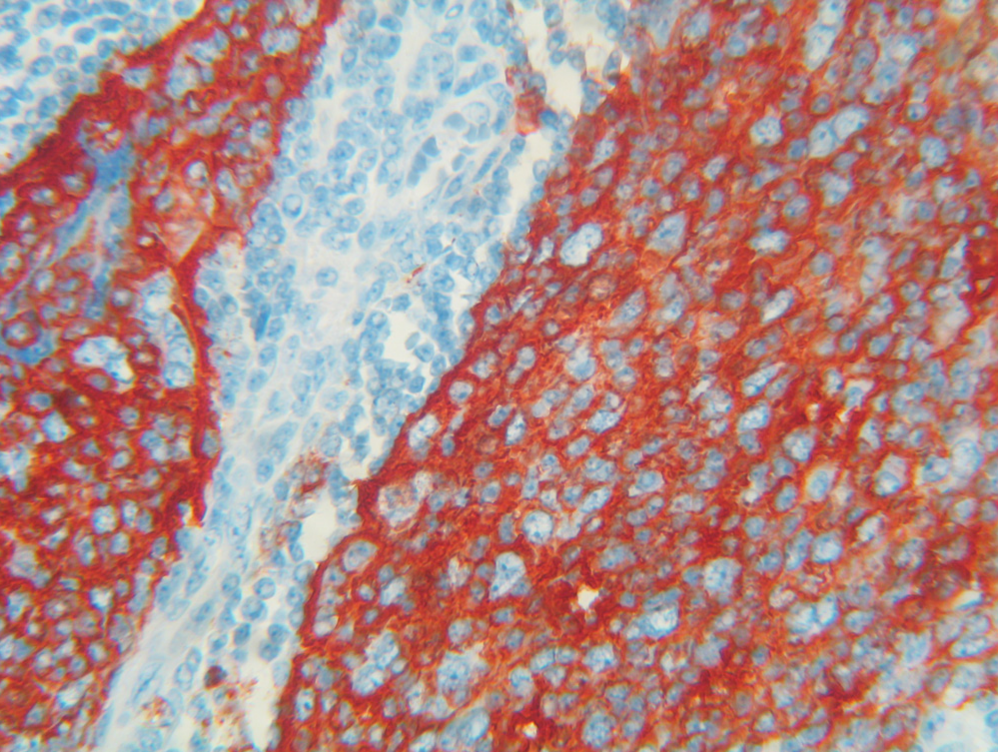
Positive staining for CD23

The follicular areas of lymph node were disappeared but residual T lymphoid tissue was generally present. Immunologically, the tumor cells showed a close resemblance to the normal follicular dendritic cells with consistent expression of CD21 [C3d complement receptor] (Figure 2) and CD23 (Figure 3). Tumor cells were negative for cytokeratin, CD45, CD20, CD3, CD30, CD68, CD35 and CD1a; Ki-67 was positive in about 15% of the cells. The final diagnosis was a follicular dendritic cell sarcoma. Other tissue specimens from nasopharynx, pyriform sinus and postcricoid area were free from malignant involvement.

### Clinical data

Routine hematological parameters showed hemoglobin 13.1 g/dl, WBC 5.9 × 10^9^/l (53% polymorphonuclear cells, 39% lymphocytes, 8% monocytes), platelets 214 × 10^9^/l, all the other biochemical parameters were normal. Hepatitis B and C, HIV serology were negative, EBV serology showed no IgM antibodies but positivity for IgG anti VCA and IgG anti EBNA.

Bone marrow aspiration and trephine yielded a normal hemopoiesis. A total body computed scan was made showing multiple hepatic angiomas (confirmed by MRI scan) and small subpleural fibrocalcific residuals, the rest of the exploration was normal. The patient also underwent an echocardiography that disclosed a good cardiac function with normal ejection fraction.

Postoperative adjuvant chemotherapy was administered; the patient received five courses of COP+PEG-liposomal doxorubicin according to the following schedule: Cyclophosphamide 750 mg/m^2 ^i.v. (day 1), Vincristine 1.4 mg/m^2 ^(day 1), PEG-liposomal doxorubicin (Caelyx) 40 mg/m^2 ^i.v (day 1) and Prednisone 100 mg po days 1–5, every 3 weeks.

## Results

No hematological toxicity was reported, but WHO grade 3 skin toxicity was observed. In particular the patient after the fourth cycle developped a palmar erytrodysesthesia with painful erytema that was attributed to PEG-liposomal doxorubicin and consequently we reduced at 70% the dose of Caelyx in the last course of treatment. The erytrodysesthesia was transitory and successful treated with mild dose of prednisone per os.

**Figure 4:**
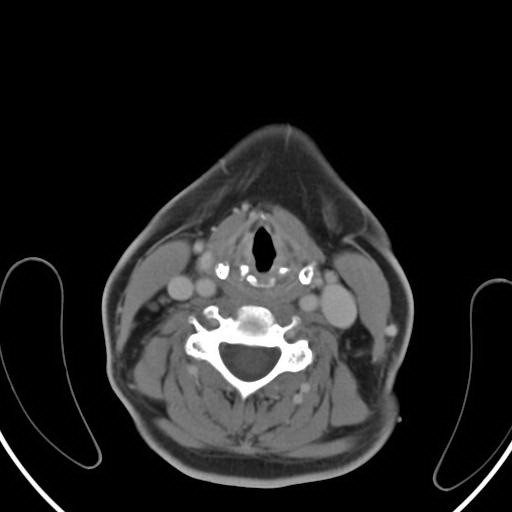
Cervical region CT scan, one month after chemotherapy, negative for residual mass

**Figure 5:**
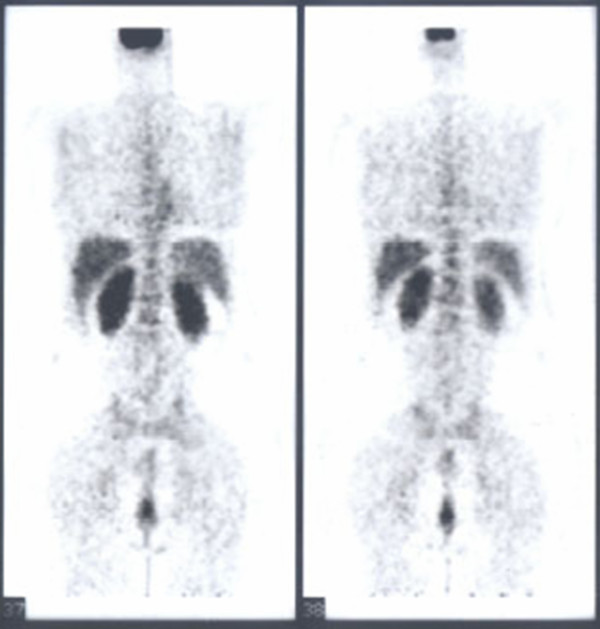
Total body 18-FDG PET three months after chemotherapy, showing no residual tumor activity

A total body CT scan performed one month after the last course of chemotherapy was negative for lymphoadenophaty (Figure 4) and previous subpleural fibrosis was not modified. Three months after chemotherapy, the patient also underwent total body 18-F-FDG PET/CT that showed no residual tumor activity (Figure 5). The patient is disease free at 5 years after the diagnosis with CT scan negative.

## Discussion

Follicular dendritic cell sarcoma is a rare neoplasm that can involve lymph nodes or extranodal sites [2,4,5]. Once FDC sarcoma is suspected from the histologic findings, immunohistochemical stains for follicular cell differentiation must be performed to avoid the potential for misdiagnosis. CD21 and CD35, directed respectively against the C3d and C3b receptors, together with CD23 and clusterin are the most widely used markers, demonstrating follicular dendritic cells differentiation and should help in their recognition [2,6-9].

FDC sarcoma was considered an indolent tumor with low tendency of recurrence or metastasis; but recent larger reports with longer follow up have showed that FDC sarcoma is more aggressive tumor and should be considered an intermediate-grade malignancy. Chan et al. have reported that at least 40% of documented FDC sarcomas have recurred and 25% have metastasized with a mortality rate of 16.7% [2,10].

Because this significant recurrent and metastatic potential it is reasonable that resected localized disease may be prevented from recurrence by adjuvant radiotherapy or chemotherapy [11-13].

In our case report, the patient underwent surgical excision and postoperatively was given five cycles of COP plus Caelyx, utilizing cyclophosphamide, vincritine, prednisone and pegylated liposomal doxorubicin.

PEG liposomal doxorubicin is an alternative preparation of doxorubicin, which has been shown to have reduced cardiotoxicity and is characterized by a very long circulation half-life, favorable pharmacokinetic behaviour and specific accumulation tumor tissues [14,15]. PEG liposomal doxorubicin has already shown activity in soft-tissue sarcoma, head and neck cancer, multiple myeloma, aggressive non-Hodgkin's lymphoma.

Based on these encouraging experiences and in consideration of the higher response rate, shorter time to response and less toxicity obtained with PEG liposomal doxorubicin also in the treatment of AIDS-related Kaposi's sarcoma [16], compared with conventional doxorubicin, we decided to treat our patient with COP plus Caelyx regimen, even if she had no particular cardiac risk. No hematological or cardiac toxicity was registered and among the other extra hematological effects only palmar erythrodysesthesia was worthy of mention. The patient is alive and event free at 5 years after diagnosis.

In the literature treatment modality of FDC sarcoma varied widely although surgical resection was often included. With a median follow up of 18 months, about 40% of the cases recurred and the 5 years recurrence-free survival was 27.4%, further the role of chemotherapy and radiotherapy in the treatment of this neoplasm is not yet clearly defined [2,10]. Clinical findings, histology and immunophenotype of our case appear to be typical of FDC sarcoma but the complete resection achieved by surgery and the use of adjuvant COP plus PEG liposomal doxorubicin, regimen ever reported to our knowledge to treat this tumor, and the very good response at 5 years of follow-up show some issues of interest.

A longer follow-up and further studies are warranted to determine whether this combination treatment may be useful in the management of FDC sarcoma, at least in localized nodal disease, once it is identified and well characterized by the appropriate application of immunohistochemical staining.
